# A Novel *N*-Input Voting Algorithm for *X*-by-Wire Fault-Tolerant Systems

**DOI:** 10.1155/2014/672832

**Published:** 2014-10-19

**Authors:** Abbas Karimi, Faraneh Zarafshan, S. A. R. Al-Haddad, Abdul Rahman Ramli

**Affiliations:** ^1^Department of Computer Engineering, Faculty of Engineering, Arak Branch, Islamic Azad University, Arak, Markazi, Iran; ^2^Department of Computer Engineering, Faculty of Engineering, Hamedan Branch, Islamic Azad University, Hamedan, Iran; ^3^Department of Computer and Communication Systems Engineering, Faculty of Engineering, UPM, 43400 Selangor, Malaysia

## Abstract

Voting is an important operation in multichannel computation paradigm and realization of ultrareliable and real-time control systems that arbitrates among the results of *N* redundant variants. These systems include *N*-modular redundant (NMR) hardware systems and diversely designed software systems based on *N*-version programming (NVP). Depending on the characteristics of the application and the type of selected voter, the voting algorithms can be implemented for either hardware or software systems. In this paper, a novel voting algorithm is introduced for real-time fault-tolerant control systems, appropriate for applications in which *N* is large. Then, its behavior has been software implemented in different scenarios of error-injection on the system inputs. The results of analyzed evaluations through plots and statistical computations have demonstrated that this novel algorithm does not have the limitations of some popular voting algorithms such as median and weighted; moreover, it is able to significantly increase the reliability and availability of the system in the best case to 2489.7% and 626.74%, respectively, and in the worst case to 3.84% and 1.55%, respectively.

## 1. Introduction

One of the fault tolerance mechanisms in software and hardware systems is fault masking in which voting algorithms are used as the principle basis in increasing the systems dependability. In these systems, *N* redundant hardware modules or software versions perform similar operations in parallel and their outputs are being voted for masking the effect of one or more run time errors [1]. Finally, the data which is more probably accurate based on the used voting algorithm will be chosen as the system output. The element performing this operation is called a voter [1]. Voting is an important operation in multichannel computation paradigm and realization of ultrareliable and real-time control systems that arbitrates among the results of *N* redundant variants which are used in various applications such as safety critical computer control systems (e.g., flight control systems, nuclear power station, and military applications), high reliable applications (e.g., file server applications and call processing applications) [1], highly available systems (e.g., distributed database and ad hoc networks) [2], and distributed systems (e.g., clock synchronization and Byzantine agreement). In some of these applications, in order to decrease the complexities and simplify the operations, the number of redundant modules is regarded as being small, mostly 3 or 5 modules; however, in some situations voting with fairly large number of inputs is required [3, 4]. Some examples are in image processing filters in which, during each pass, the value of pixels may be replaced with the values determined from the voting on the predetermined values of the neighboring points [5] or in data fusion originating from a large number of sensors [6, 7], implementation of cellular automata and neural networks [1, 8], diagnostic functions in parallel, and distributed systems and systems alike [3, 9–11]. Consequently, we need such voting algorithms which can vote on large-numbered inputs and such voting must be reasonable in respect to computation complexity [12], reliability [13], availability [14, 15], and other dependability criteria. So far, different voting algorithms have been proposed which have been efficient in some aspects more than the others based on their particular features, for example, *M*-out-of-*N* [16, 17], majority, plurality [3, 18], different weighted methods [1, 19, 20], median [5, 9], predictive [13, 21], and smoothing algorithms [22].

In most voting algorithms, the algorithm will be able to generate output (agreement) on the condition that at least the certain numbers of voter inputs which are the outputs of redundant variants generate exactly the same results. Such an agreement is known as exact voting. While in inexact voting, agreement means that the multiple results are not exactly the same, but their difference from each other is smaller than a predefined application specific threshold. In many applications, the results of multiple redundant modules may not be completely identical even in a fault-free environment. Examples include redundant sensor outputs which are read by digital computers or the output of diversely implemented software programs which handle floating-point calculations. In such applications, an inexact voting is required [4]. As a result, most voting algorithms are either inexact or designed so that they can operate regardless of the agreement type.

In this paper, we introduce a novel voting algorithm for *N*-input systems which is able to vote on disordered inputs. This algorithm is provided in both exact and inexact agreement types and software implemented in different scenarios of fault-injection and faulty modules. The analysis of the results shows that, beside its appropriateness for the systems with large number of inputs, the novel algorithm has better performance in both exact and inexact scheme in comparison with similar algorithms.

This paper is organized as follows: in Section 2, the related works and the current voting methods will be introduced.  Section 3 deals with introduction of the new algorithm in both exact and inexact types and provides relevant examples. In Section 4, the model being used in voter simulation and the evaluation criteria will be introduced.  Section 5 deals with the simulation results, analyzing it in both exact and inexact types through extracted plots and tables. Finally, conclusions and future works will be stated.

## 2. Related Works

Voting algorithms have been extensively applied in situations where choosing an accurate result out of the outputs of several redundant modules is required [3, 9]. Most of proposed voting algorithms in literature are structurally able to vote in systems either with small or large number of voter inputs, although their performance is often simply examined in small conditions. One of the most applicable voting schemes is *N*-input majority voter which produces an output among variant results, where at least (*N* + 1)/2 variant results agree. *N*-input plurality voter [3] is the relaxed form of majority voter and implements *M*-out-of-*N* voting, where *M* is less than a strict majority (e.g., 2-out-of-5 or 3-out-of-7 voting). Plurality voters normally have an odd number of variants, so that a tie does not occur. If adaptive voting is used which degrades the system to an even number of variants, then the algorithm must switch to majority voting to avoid a tie [4]. Both majority and plurality algorithms are particular forms of *M*-out-of-*N* algorithm in which agreement will be obtained if the minimum *M* inputs out of the *N* voter inputs agree. This voting method is a suitable choice for the systems where the number of voter inputs is large. In [23, 24], the exact forms of the above-mentioned algorithms along with their operation and time complexity for small and large number of inputs are considered. Other types of exact voters are presented in [25]. The exact types of voters are more appropriate for hardware applications. However, as described in Section 1, inexact voting is practically more suitable in real conditions. In [18], some of the most popular inexact voters for *N*-input systems such as median, inexact plurality, inexact majority, and weighted average voter have been introduced.

The operating method of majority and plurality voters in inexact agreement is the same as the exact one; whereas in inexact agreement, if the distance measurement between the voter inputs is smaller than the predefined and certain value, known as voting threshold, they are considered to be the same inputs. For *M*-out-of-*N*, we can similarly consider an inexact scheme [16, 23]. The median voter is a mid-value selection algorithm. The most significant limitation of this algorithm is that the number of the voter inputs is assumed to be odd [18]. In weighted average algorithm, the weighted mean of the input values is calculated as the voting result. The weight value is assigned to each voter input in various methods, for example, [1, 10, 11, 20, 26–29]. Then, calculated weights, *w*_*i*_, are used to provide voter output, *y* = ∑*w*_*i*_ · *x*_*i*_/∑*w*_*i*_, where *x*_*i*_ are the voter inputs and *y* is the voter output. In this method, the voting results may be clearly different from input values. The main problem with all the weighted methods is the increasing in the complexity of weights and voter output calculations while the number of voter inputs increases. Moreover, weighted and median voters are unable to produce benign outputs which can lead to hazardous events particularly in safety critical systems. Such other inexact voters as smoothing [22], linear, first order, and three-domain voters are all the extended forms of inexact majority algorithm in which when there is no agreement between input values, the voter history record is used to guess the probable output for this voting cycle [26, 30]. Afterward, this value is compared with each voter input and the closest voter input to this probable correct output is considered as voter output, on the condition that their difference is less than a predetermined certain threshold; otherwise, the voter fails to produce answer and the algorithms can be modified to produce benign output; that is, conduct the system to a fail-stop or fail-safe situation [31]. In this paper, we have utilized the idea of these algorithms to provide a novel voter suitable for *N*-modular systems.

## 3. The Advanced *M*-out-of-*N* Algorithm

This algorithm is an extended form of *M*-out-of-*N* threshold voting algorithm [32, 33] to which a particular acceptance test is added. This acceptance test operates based on this assumption that a discontinuity among the consecutive output results of the voter is index of an error occurrence. This assumption is valid in many real-time control applications in which there are feedback control and periodic calculations [13, 22]. The novel algorithm has no limitation due to odd or even number of its inputs and there is no need to sort the voter inputs. It is also assumed that the first voting cycle is successful and the output is available for this cycle.

In this voting algorithm, *x*_*i*_ are inputs of the voter where *i* = 1,2, 3,…, *N* and their nonnegative real weights are demonstrated as *v*_*i*_ (∑*v*_*i*_ = *V* and *N* = *V*); therefore, each voter input can be introduced as (*x*_*i*_,*v*_*i*_) and we need *p* = ⌊*V*/*t*⌋ storage spaces or slots for different inputs object_1_, object_2_,…, object_*p*_ where each object_*j*_ has an associated voter total tally_*j*_. In this algorithm, in case that the total weight of the agreed inputs get to *t* (*t* = *M*, if the weights are equal to 1), then the voter can produce output.

The structure of advanced *M*-out-of-*N* algorithm is described as shown in Algorithm 1.

In the first line of the algorithm, after determining the number of slots, the first input pair is saved in the first slot and other tally_*j*_s are assigned to zero. In the second line, the input objects *x*_1_, *x*_2_, *x*_3_,…*x*_*n*_ are investigated in turn. Later, next voter input (next *x*_*i*_) is compared with the saved object(s) in the slots. In inexact voting, if the measured distance is smaller than the voting threshold, *τ*, the tally of agreed slot is added to *v*_*i*_ (line 4). If exact voting is desired, the term |*x*_*i*_ − object_*j*_| ≤ *τ* needs to be replaced with *x*_*i*_ = object_*j*_ in lines 3 and 17. If input *x*_*i*_ is not in agreement with any of object_*j*_s and less than *p* objects are saved in *p* slots and there is an empty slot (a slot whose tally value is 0), (*x*_*i*_, *v*_*i*_) is saved in it (line 6). In line 7, if all slots are occupied, then the minimum weight of the previous voter inputs (tally_*j*_s) must be found (known as tally_*k*_). If *v*_*i*_ ≤ min⁡, then the new input *x*_*i*_ is ignored and all the saved tallies are decreased by *v*_*i*_ (line 9); otherwise, the vote tallies are decreased by min and (*x*_*i*_, *v*_*i*_ − min⁡) is replaced with one of the objects which has the vote tally 0 (line 10). Therefore, in the first pass of the algorithm, the *x*_*i*_, which probably is the consensus result, is found. Second pass of the algorithm (lines 16–20) finds out if at least *M* inputs agree with resulted value in previous pass. To do so, the weights of the voter inputs whose objects are in agreement with the resulted value in first pass, are added together and the sum is saved to the related tally_*j*_. As soon as a tally_*j*_ gets to *M*, the algorithm is stopped and the related object_*j*_ value is announced as voter output. If the algorithm cannot obtain any agreement in first and second passes, the probable voting correct result has to be calculated in lines 21–25. So, the closest input to the last successive voter output is chosen as the probable correct voting result, on the condition that the measured distance is smaller than a predetermined and certain value called advanced threshold, *γ*; otherwise, voter fails to produce output. In this case, we can program voter to fail safe and generate benign output. Benign outputs are attributed to the cases where the voter is not able to generate output, and if such outputs are not distinguished from the others, they can lead to incorrect system result which especially in safety critical systems can cause hazardous events. In all the voting algorithms using threshold, selecting the appropriate value of threshold is critical. Although these values are arbitrary, the performance of the algorithm is promoted only if there is some available information about the probable size of discontinuity among the consecutive outputs during system mission in order to select the fair threshold value [22].

In Table 1, the results of 8 consecutive voting cycles of the inexact advanced *M*-out-of-*N* algorithm and inexact *M*-out-of-*N* threshold algorithm are shown accompanied with voter inputs with the assumptions *N* = 5, *M* = 3, *τ* = 0.2, *γ* = 1, and *v*_*i*_ = 1.

The original input data of each voting cycle has been shown in the first column, the unordered voter inputs are presented in columns 2–6, and the voting result is mentioned in the last column. If the difference between the voting result and the original input data is more than an arbitrary threshold called accuracy threshold, *ϵ*, which is assumed to be 0.5, the output will be regarded as incorrect. The accuracy threshold is a parameter in test environment and is merely a measure to classify correct and incorrect results. In Table 1, the cases, for which the voter has failed to vote or has produced incorrect outputs, are highlighted.

In Table 1, clearly, in cases where *M*-out-of-*N* algorithm is unable to produce the output, the novel algorithm has often produced correct results.

## 4. Performance Evaluation

### 4.1. Evaluation Method

**Figure 1:**
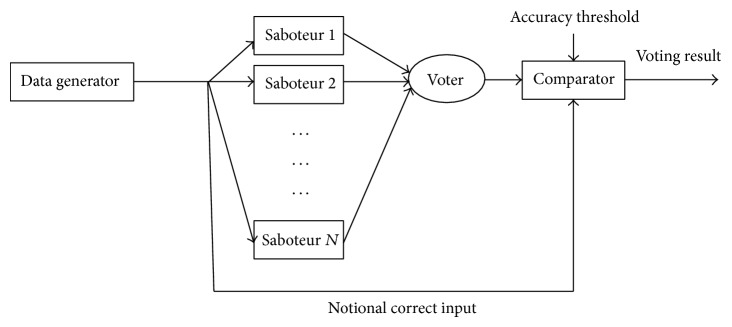
The experimental test harness.

To evaluate the dependability of the novel algorithm, the extended form of the test harness introduced in [31] is applied to simulate the voting system, with this assumption that the voter is used in a cyclic system in which there are relations between the correct results of a cycle with the next cycle. This assumption is valid in many real-time control systems. The input data of the system are considered as numerical nonnegative values and it is hypothesized that faults can lead to the errors whose effects might be revealed in the form of a difference between the output values of the modules. The differences less than the voting threshold are ignorable in inexact voting. For the purposes of the results reported here, the issues associated with ensuring synchronization of the inputs to the voter are ignored [13, 22]. In the simulation, we assume *τ* = 1, *γ* = 2, and *ϵ* = 1. In Figure 1, the structure of the experimental test harness is illustrated. This structure is widely common in evaluating the voting methods (e.g., see [22, 34, 35]).

This model is made up of an input data generator, a repeater, *N* saboteurs, a voter, and a comparator. In simulation, our focus is on voter performance; hence, other elements such as data generator, the connection links, and comparator are considered fault-free. In each voting cycle, the data generator generates the notional correct input by using a polynomial which is round |100sin⁡(*t*) + 100|. Identical value of *t* is zero and is periodically increased by 0.01 in next cycles. The repeater copies the notional correct input to *N* saboteurs. Saboteurs are programmed so that each one injects a random error value between [0–10], produced by using the uniform distribution function to their inputs. This action resembles the practical conditions in which the module outputs become erroneous because of various reasons, for example, noises, floating-point calculations, and inner modular faults. We consider different scenarios of error injection to saboteurs, from the case where all modules are safe (complete agreement) to the extent that there is only one faulty module or two faulty modules or all modules are faulty (complete disagreement). Afterward, the voter arbitrates among the module outputs. To distinguish the correctness or incorrectness of the voting results, the output of the voter is compared with the notional correct input. If their difference is less than the accuracy threshold, the voter output is correct; otherwise, it is incorrect. This assumption is valid in many real-time applications in which discontinuity between the consecutive correct results is small [36]. If the voter fails to produce an agreement, benign output can be produced [34]. Based on the above-mentioned issues, the following criteria are defined for analysis and comparison of voting algorithms.

*N*_*t*_ is number of total runs of a voter in a given test. In this research *N*_*t*_ is 10000 times for each *M*.
 
*N*_*a*_ is number of agreed results among *N* output. A voter which produces more agreed results among its total runs can be interpreted as an available voter. Availability is defined as the ratio of agreed voter results to the number of voting actions: *A* = *N*_*a*_/*N*_*t*_. Thus *A* ∈ [0-1] and ideally *A* = 1.
 
*N*_*c*_ is number of agreed-correct results. A voter which produces more correct results among its total outputs can be interpreted as a reliable voter. Reliability is defined as the ratio of correct voter outputs to the number of voting actions: *R* = *N*_*c*_/*N*_*t*_. Thus, *R* ∈ [0-1] and ideally *R* = 1 [13, 34].
 
*N*_*ic*_ is number of agreed but incorrect results. Because the minimum number of agreed but incorrect agreements in a given voter is desirable, the safety criterion can be defined as *S* = 1 − *N*_*ic*_/*N*_*t*_. Thus, *S* ∈ [0-1] and ideally *S* = 1 [14, 37, 38].
 
*N*_*d*_ is number of disagreed results among *N* outputs (or the number of benign outputs).

Based on the above definitions clearly *N*_*t*_ = *N*_*c*_ + *N*_*ic*_ + *N*_*d*_.

### 4.2. Simulation Results

The comparisons of voting results for selected scenarios based on defined criteria in Section 4.1 are presented in this section. To have a comprehensive analysis of the proposed voter, the simulation has been implemented in two different cases of *N* and *M*. First, we consider *N* as a constant value and variable *M* and second, variable *N* and constant value of *M* are determined. In both cases, exact and inexact agreements are studied separately. The simulation has implemented for *N* = 4, 8, 16, 32, 64, and 128. To fulfill the purpose of this paper, the results of large values of *N*, that is, 64 and 128, are stated.

#### 4.2.1. Variable *M* and Constant *N*

**Figure 2:**
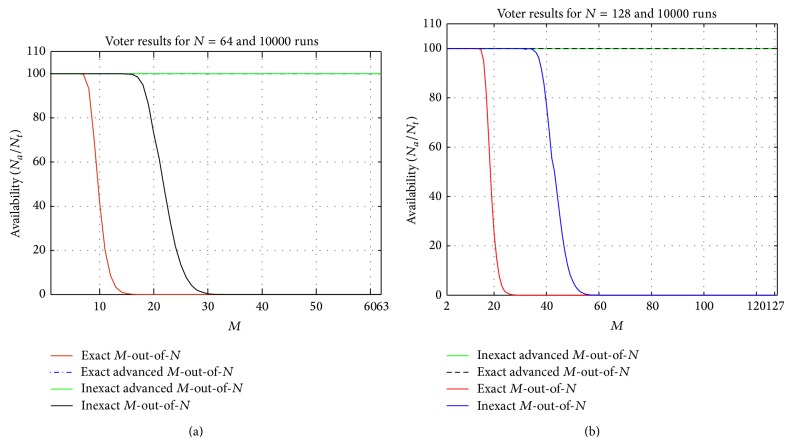
The percentage of agreed results of voting algorithms for (a) *N* = 64 and (b) *N* = 128.

**Figure 3:**
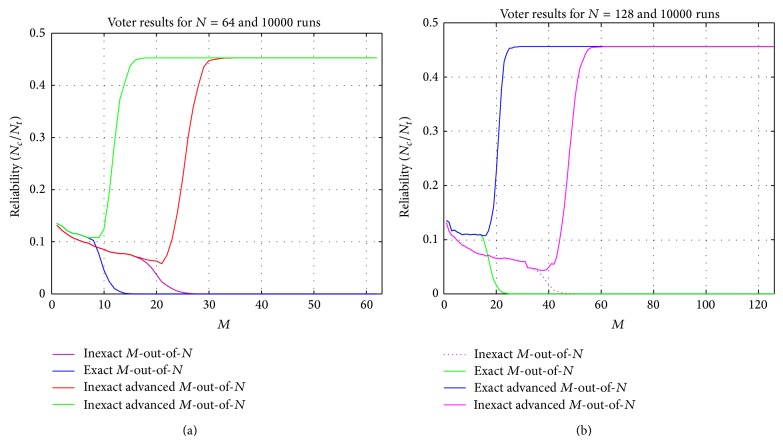
The percentage of correct agreements (reliability) of voting algorithms for (a) *N* = 64 and (b) *N* = 128.

In the plots of Figures 2 and 3, the result of 10000 runs of *M*-out-of-*N* and advanced *M*-out-of-*N* algorithms are illustrated. In Figure 2, the *x*-axis and *y*-axis, respectively, display the changes of *M* (from 2 to *N* − 1) and the percentage of agreed outputs (availability) of voters for exact and inexact agreements. The plots clearly show that the availability of the novel algorithm for both exact and inexact agreements and for *N* = 64 (Figure 2(a)) and *N* = 128 (Figure 2(b)) is 100 percent; meanwhile, in *M*-out-of-*N* algorithm, not only this value is less, but also, for some values of *M*, it is zero. The plots demonstrate that the novel algorithm has improved the availability of a system with *N* = 64 to 560.5% for exact agreement and 189.1% for inexact agreement and in a system with *N* = 128 to 202.02% for exact and 626.74% for inexact agreement.

Similarly, plots of Figure 3 show the reliability of exact and inexact voting algorithms for different values of *M* (from 2 to *N* − 1). In plots of Figures 3(a) and 3(b), the reliability of voting algorithms for *N* = 64 and *N* = 128 are compared, respectively. The reliability of novel algorithm is clearly much more than *M*-out-of-*N* voter in both exact and inexact schemes. Moreover, in the cases that this value is zero in *M*-out-of-*N* algorithm (due to its nonproducing agreements which is described in Figure 2), the new algorithm has improved the reliability of the system with *N* = 64 in exact and inexact schemes to 2088.2% and 956.79%, respectively, and with *N* = 128 in exact and inexact schemes to 1303.6% and 2489.7%, respectively.

As mentioned in Section 4, our experimental results are not limited only to *N* = 64 and 128; accordingly, we have also studied the plots similar to Figures 2 and 3 for smaller *N* values in which the same mentioned results were achieved. Hence, it can be concluded that the new algorithm has superiority over *M*-out-of-*N* algorithm in regards to availability and reliability criteria in both exact and inexact schemes.

#### 4.2.2. Variable *N* and Constant *M*

**Figure 4:**
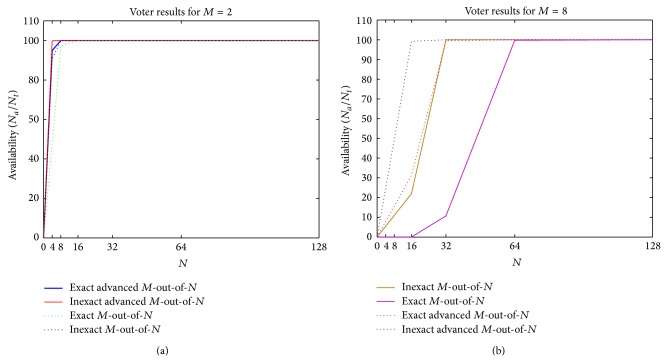
The percentage of agreements (availability) of voting algorithms for (a) *M* = 2 and (b) *M* = 8.

**Figure 5:**
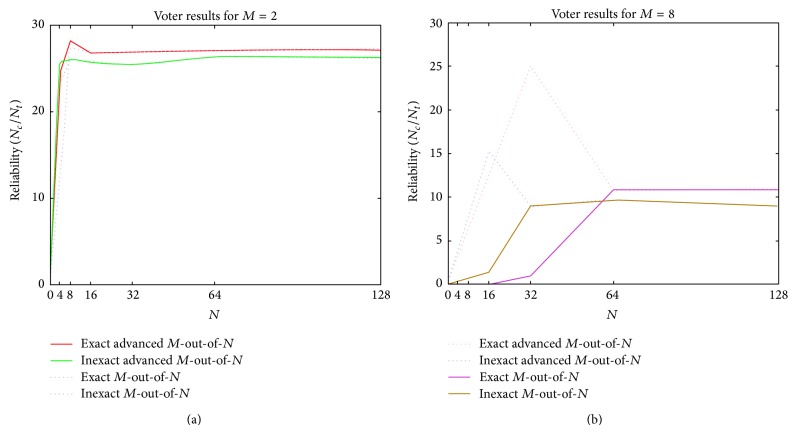
The percentage of correct agreements (reliability) of voting algorithms for (a) *M* = 2 and (b) *M* = 8.

We did not only rely on the simulation results presented in Section 4.2.1 and for more clarification of the results, the cases where *M* is constant are also considered. The simulation results of *M*-out-of-*N* and advanced *M*-out-of-*N* algorithms in exact and inexact cases, for *M* = 2 and *M* = 8, have been presented in Figures 4 and 5. The availability of voting algorithms for *M* = 2 when *N* = 4,…,128 and *M* = 8 when *N* = 8,…,128 is, respectively, illustrated in Figures 4(a) and 4(b). The comparisons show that the availability of the new algorithm for *M* = 2 has improved to 9.15% and 1.55%, respectively, in exact and inexact schemes. Similarly, in new algorithm, the availability has increased in exact and inexact schemes to 57.21% and 23.99%, respectively. However, this increase is more tangible for *M* = 8.

Similarly, the reliability of *M*-out-of-*N* and advanced *M*-out-of-*N* algorithms in both exact and inexact schemes are compared with each other for *M* = 2 and *M* = 8, respectively, in Figures 5(a) and 5(b). As it is clear, the new algorithm has improved the reliability of the system for *M* = 2 and *N* = 4,…,128 (Figure 5(a)) to 8.47% and 3.84%, respectively, in exact and inexact schemes and for *M* = 8 and *N* = 8,…,128 (Figure 5(b)) to 160.59% and 47.66%, respectively, in exact and inexact schemes in comparison with *M*-out-of-*N* voter.

It can be concluded from what was described in Sections 4.2.1 and 4.2.2 that the performance of our new proposed algorithm in both exact and inexact schemes has superiority over the other similar voting methods in respect to generating more agreements (availability) and the percentage of correct agreements (reliability), especially in the cases in which the number of voter inputs is large.

It is worth mentioning that in voting, so far, as the number of *M* is decreased, the probability of agreement is increased and it is provable, that is, the reason of close results of the simulation of our new algorithm and *M*-out-of-*N* algorithm in the case *M* = 2; however, the important matter in large scale systems is that a voting method can produce more agreed and correct results in condition that the value of *M* (the minimum number of agreed inputs to make an agreement) is large and it is the main focus of our proposed approach.

## 5. Conclusion

In this paper, a new voting algorithm appropriate for real-time control systems has been introduced and software implemented. The extracted results of the algorithms simulation—through random error-injections in different scenarios including complete agreement and agreement of a certain number of inputs to complete disagreement—demonstrated that the new algorithm has a higher percentage of agreements and correct results (more availability and reliability) in comparison with the similar algorithms which makes the novel algorithm suitable for the systems in which the number of voter inputs is large. Furthermore, the new algorithm has no limitation existing in some voters like median on the odd or even number of inputs and sorting the input data. In the new algorithm, contrary to different types of weighted voters, the complexity of calculations does not increase with increasing the value of *N*. Another problem of median and weighted algorithms in common is their inability to produce benign outputs, a fact which does not exist in our new algorithm. From the above-mentioned issues and the results of Section 4, it can be concluded that, for some real-time fault-tolerant applications in which more availability and reliability of system than some common voting algorithms such as median, weighted, and *M*-out-of-*N* are main resolutions, the new voting algorithm can be an appropriate solution.

In the future works, we are supposed to improve the algorithm run time speed and system dependability criteria by using a more comprehensive acceptance test and extend the new algorithm to parallel and distributed applications.

## Figures and Tables

**Algorithm 1:**
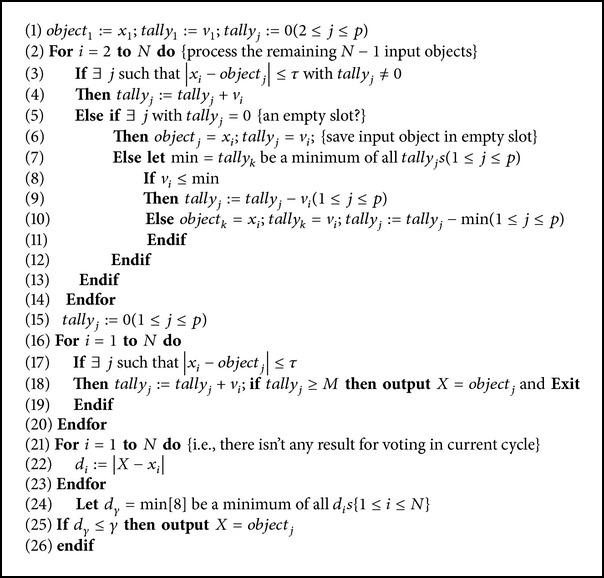


**Table 1:** The voters results for 8 consecutive voting cycles.

Sample	*X*_1_	*X*_2_	*X*_3_	*X*_4_	*X*_5_	*M*-out-of-*N *	Advanced *M*-out-of-*N *
1	1.1	1.3	1.3	1.1	1.5	1.1	1.1
2	2.1	2.5	2.2	2.1	3	2.2	2.2
3	2.7	2.8	3.1	8.9	3.5	**No result**	2.7
4	3.8	4.2	4.1	3.5	5	**No result**	3.5
5	4.2	5	5.5	4.7	5.1	**No result**	4.2
6	5.9	6.3	6	6.2	7	6	6
7	6.5	7.2	6.5	7.2	7.8	**No result**	6.5
8	8.6	8.2	7.8	8	8.1	**No result**	**No result**
